# To Be or Not to Be; That Is the Question

**Published:** 1885-08-20

**Authors:** 


### "TO BE OR NOT TO BE ; THAT IS THE QUESTION."

Not only our domestic journals but those from abroad have latterly taken up an issue raised by certain members of the Medical profession in respect to the proposed International Congress, and the correspondence of the views presented in the Medical Times and Gazette, in the Medical Press, and in the British Medical Journal, affords a most striking coincidence, which smacks of American prejudice against the new organization. We have thus far looked on quietly at the agitation respecting the change of programme for the organization of an International Congress, at Washington, in 1887. We have noted the exhibition of partizan feelings by various medical journals, and also the manifestations of good judgment by others that view the whole situation from a purely medical standpoint. It is to be hoped that the differences shall be reconciled without any detriment to the cause of Medicine, or to the status of the Profession in the estimation of foreigners, who are expected to co-operate with American physicians in this reunion. Notwithstanding the strife and bitterness engendered amongst the medical men of this country, we know by the results of great excitement connected with political agitation in the United States that all may blow over and leave the waters calm in the course of time.

As to the merits of the controversy, all depends upon the relation of a principal to the agent in transacting business, and how far delegated powers involve the responsibility of the body that authorizes a portion of the members composing it to represent its interests. In this instance, the authority of the American Medical Association, at the meeting of 1884, was given to a committee of seven to invite the International Congress to hold its meeting in 1887 in Washington, and, in case of acceptance, to make the preliminary arrangements for its organization. It is held that, in the discharge of the work entrusted to this committee, there was a disregard of the recognized attitude of the Association towards New Code men, by assigning some of them to prominent places in the proposed organization of the Congress, and that there was a notable departure from propriety in confining the appointments to a comparatively limited section of the United States. This conviction, added to other motives for changing the basis adopted by the original committee, led to the appointment of a committee representing all portions of the country and every department of the profession, to co-operate with the first committee, having certain definite instructions as to their future proceedings. Although there was considerable opposition manifested to the principles involved in this reconsideration of its previous action by the Association, it was only after the meeting of the committee at Chicago, under the new order of things, that this opposition assumed a distinct form, and led to the positive refusal of some good and true men to fulfil the parts assigned them in the reformed programme. It is held by those who decline to co-operate in the present organization that "these changes are inconsistent with the original plan, and detrimental to the interests of the Medical profession in America, and of the International Medical Congress." But others, who adhere to the revised programme, insist that all has been done in strict accordance with the principles heretofore avowed, and acted upon, by the American Medical Association, and which look to the honor and best interests of the medical profession.

That other considerations than a high regard for the status of American medicine may have prompted those who have seceded cannot be urged, in view of the published resolutions of those parties; but the favorite recourse for the redress of grievances throughout the world has been to act upon the principle of the "Man who cut off his nose to spite his face," and we have a signal illustration of this suicidal policy in the action of the "rule or ruin" element of the medical profession in this country at present.

If the parties in Philadelphia, Boston, Baltimore, Washington, and Cincinnati, with isolated instances in other cities, who have withdrawn from any connection with the International Congress, had good and sufficient reasons for their proceeding, it would have been proper to set forth in clear and unmistakable terms the ground of exception, and not left their brethren throughout the land to guess at some undefined cause of dissatisfaction. Should it turn out that this action is based upon personal considerations touching the chairman and secretary of the new committee, rather than objections to the fundamental principles involved in the changes inaugurated by the American Medical Association at New Orleans, it certainly devolved upon them to give some expression of their convictions in this respect. The impression that any association, with objectionable individuals in an official capacity, was to be remedied by refusal to serve under the appointments made by the committee as a whole does not comport with the high regard for the great interests involved, and grows out of a misconception of the means to be adopted for correcting such a grievance, if there be a just cause of complaint.

It is greatly to be regretted that the Congress is to be deprived of the influence of the names of those who have been selected by the committee as eminently well fitted by reason of their reputation to give eclat to the organization, and it is still more to be regretted that the gentlemen have seen fit to associate themselves together to advertise to the world their dissatisfaction with the proceedings of the new committee, and their withdrawal from any part or responsibility in the organization of the International Congress; nevertheless, we congratulate the country that they may be replaced by others equally able, equally learned, and equally zealous for the progress of American medicine The defection of friends is always a source of embarrassment to any cause, and good men should not indulge in gloomy forebodings with regard to this movement without giving some reason for their course which will commend itself to the good sense of all who are interested in this International organization. Had these members of the profession never been mentioned in connection with the positions assigned them, their services could be well dispensed with; but they have been put forward in a way to cause their desertion to injure a much cherished undertaking, and it is hoped the seceders may reconsider their action and go to work like good and true men—as we know most of them to be—to make the Congress a source of honor and pride to our professional countrymen. But should they persist in their defection, and remain sulking in their tents, there will be no other alternative left the committee of organization but to call upon others, who appreciate the importance of the work, to fill their places, and to push forward the preparations for the meeting of the Congress.

The fact that others will readily be found to worthily discharge the duties attached to their several positions, ought not to allow their withdrawal to lessen the confidence of the profession in the working of the programme upon the basis adopted by the American Medical Association. It will bring to the front meritorious colleagues, who have been kept in the background by the prominence given to personages who are supposed to have superior claims to consideration, but who, really, have no higher order of qualifications in their respective departments than many others unknown to fame. The medical profession in this country has no royal orders or princely chiefs, and preferment is alike open to all. The number of eminently qualified physicians is not, by any means, limited to those who have declined the places tendered to them, and there are many others in our ranks who can do honor to the American Medical Association by representing her in the International Congress. This will demonstrate to the medical world the advancement that has been made in our country by medical men generally, and not rest our claims to consideration upon the fame of any one hundred representative men ; and it will surprise the admirers of greatness to learn how completely their places can be filled by others who will prove themselves competent for the work.

